# m7G-Associated subtypes, tumor microenvironment, and validation of prognostic signature in lung adenocarcinoma

**DOI:** 10.3389/fgene.2022.954840

**Published:** 2022-08-10

**Authors:** Guangyao Wang, Mei Zhao, Jiao Li, Guosheng Li, Fukui Zheng, Guanglan Xu, Xiaohua Hong

**Affiliations:** ^1^ Department of Respiratory and Critical Care Medicine, The First Affiliated Hospital of Guangxi University of Chinese Medicine, Nanning, China; ^2^ Department of Intensive Care Unit, The First Affiliated Hospital of Guangxi University of Chinese Medicine, Nanning, China; ^3^ Graduate School, Guangxi University of Chinese Medicine, Nanning, China

**Keywords:** m7G, lung adenocarcinoma (LUAD), tumor microenvironment, subtype, prognostic

## Abstract

**Background:** 7-Methylguanosine (m7G) is an important posttranscriptional modification that regulates gene expression and is involved in tumorigenesis and development. Tumor microenvironment has been proven to be highly involved in tumor progression and prognosis. However, how m7G-associated genes affect the tumor microenvironment of patients with lung adenocarcinoma (LUAD) remains to be further clarified.

**Methods:** The genetic alterations of m7G-associated genes and their associations with the prognosis and tumor microenvironment in LUAD patients were systemically analyzed. An m7G-Riskscore was established and analyzed for its performance in disease prognosis and association with patient response to immunotherapy. Expression of the model genes at the protein level was investigated through *ex vivo* experiments. A nomogram was finally obtained based on the m7G-Riskscore and several significant clinical pathological features.

**Results:** m7G-Associated genes were obtained from five LUAD datasets from The Cancer Genome Atlas and Gene Expression Omnibus databases, and their expression pattern was determined. Based on the m7G-associated genes, three LUAD clusters were defined. The differentially expressed genes from the three clusters were screened and used to further divide the LUAD patients into two gene clusters. It was demonstrated that the alterations of m7G-associated genes were associated with the clinical pathological features, prognosis, and tumor immune infiltration in LUAD patients. An m7G-Riskscore including CAND1, RRM2, and SLC2A1 was obtained with robust and accurate prognostic performance. WB and cell immunofluorescence also showed significant dysregulation of CAND1, RRM2, and SLC2A1 in LUAD. In addition, a nomogram was established to improve the clinical feasibility of the m7G-Riskscore. Correlation analysis revealed that patients with a lower m7G-Riskscore had higher immune and stromal scores, responded well to chemotherapeutics and multiple targeted drugs, and survived longer. Patients with a higher m7G-Riskscore tended to suffer from a higher tumor mutation burden. Furthermore, the m7G-Riskscore exhibited significant associations with immune cell infiltration and cancer stemness.

**Conclusion:** This study systemically analyzed m7G-associated genes and identified their potential role in tumor microenvironment and prognosis in patients with LUAD. The findings of the present study may help better understand LUAD from the m7G perspective and also provide a new thought toward the prognosis and treatment of LUAD.

## Introduction

7-Methylguanosine (m7G) is an important posttranscriptional modification of messenger RNA with an addition of methyl in the guanine (G) site of the m7G motif under the action of methyltransferase. It plays a regulatory role in various functional processes that take place throughout the life cycle of mRNA (Tomikawa, 2018). Research found that m7G is important in the regulation of efficient gene expression and cell viability (Bi et al., 2020). Increasing evidence has suggested that abnormal RNA methylation contributes to the occurrence and progression of cancers in humans (Xie et al., 2020). m7G, being one of the modifications of RNA methylation, plays a vital role in lung cancer progression. Lung cancer ranks top in terms of mortality among the types of cancer globally (Sung et al., 2021) and is characterized by high aggressiveness, strong drug resistance, and active angiogenesis. Ma et al. (2021) reported that METTL1 and WDR4, which are components of m7G methyltransferase, exhibited an increase in expression in lung cancer tissues compared with normal tissues, which was associated with poor prognosis. However, knockout of METTL1 and WDR4 decreased the potential of cancer cells in proliferation, invasion, and oncogenesis. Another study found that METTL1 advanced the translation of VEGFA mRNA dependent on m7G methylation, resulting in increased angiogenesis (Zhao et al., 2021).

Tumor microenvironment (TME) provides a condition with diverse and complex compositions, including tumor and non-tumor cells (such as fibroblasts, endothelial cells, and immune cells). It plays a role in tumor angiogenesis under the coordination of the circulatory and lymphatic systems as well as related cells. In addition, TME is important in the induction of immune tolerance and is conducive to invasion advancement and tumor progression. Active angiogenesis is the distinct feature of lung cancer and is also an important process in TME. It was reported that m7G methylation can advance angiogenesis. However, the relationship between m7G methylation and TME has been less studied. A comprehensive analysis on such a relationship can help better understand m7G-mediated cell infiltration within the TME of different lung cancer subtypes and reveal the potential mechanisms to increase the prognosis of lung cancer and provide new insight into the treatment.

In the present study, patients with lung adenocarcinoma (LUAD), the most common histological subtype of lung cancer (Shiba-Ishii, 2021; Succony et al., 2021), were subclassified into three molecular subtypes based on the expression of m7G-associated genes. Differentially expressed genes (DEGs) among the three subtypes were screened and used to further stratify the patients into two gene-clusters. The molecular characteristics, TME, and prognosis of the m7G-associated subtypes were assessed. An m7G-Riskscore prognostic for the overall survival (OS) of LUAD patients was established, and a nomogram of clinical significance was correspondingly generated. Expression of the model genes at the protein level was examined through *ex vivo* experiments. Taken together, this study systemically analyzed the relationship between m7G-associated genes and the prognosis, TME, and drug sensitivity in LUAD patients, which may be beneficial for the accurate prognosis of the LUAD patients and provide potential therapeutic targets.

## Materials and methods

### Data source

**FIGURE 1:**
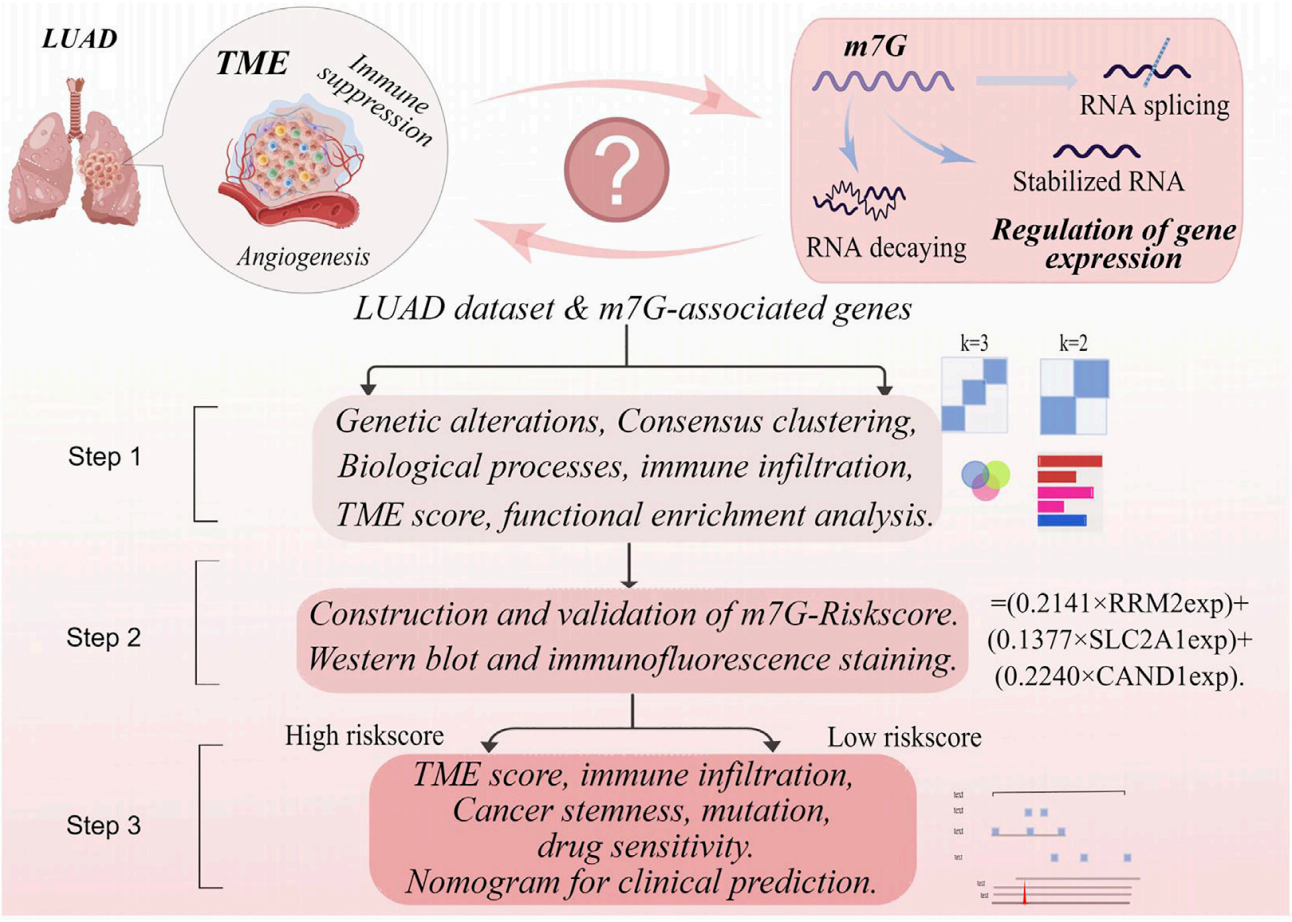
The workflow of the study.

TCGA-LUAD data in the FPKM format were downloaded on Nov. 23, 2021, from The Cancer Genome Atlas (TCGA) database (https://portal.gdc.cancer) and then converted into TPM. In the meantime, four LUAD datasets, GSE13213, GSE31210, GSE68465, and GSE30219, were obtained from the Gene Expression Omnibus (GEO) database (https://www.ncbi.nlm.nih.gov/geo/). Data from the five datasets were combined and normalized using the R software to eliminate potential batch effects (Xia et al., 2022a). Corresponding clinical data were also extracted, and samples with missing data of OS or exhibiting OS as 0 were excluded. Ethical approval for the study was waived given that all data used in the study were from public databases. The workflow of the study is displayed in Figure 1(By Figdraw, ID:YPPUY42f39).

### Genetic alterations of m7G-associated genes in LUAD

m7G-Associated genes (*n* = 29) were obtained from the published review of Tomikawa (2018). Detailed information is shown in Supplementary Table S1. Somatic mutation occurs in case of DNA damage during cell activity or DNA replication error during cell division without correct repair. It is associated with aging and disease onset, whereas cancer is the most well-known (Martincorena and Campbell, 2015; Milholland et al., 2015; Li et al., 2021a). Here, R 4.1.1 was applied to visualize the genetic alterations of the 29 m7G-associated genes in LUAD.

### Consensus clustering analysis and clinical prognosis

Consensus clustering analysis based on gene expression matrix is conducive to the diagnosis and treatment of cancer (Yu et al., 2015). Here, the R package “ConsensusClusterPlus” (Wilkerson and Hayes, 2010) was used to perform consensus clustering analysis based on the expression of m7G-associated genes to divide LUAD samples into different molecular subtypes. Reduced intergroup correlation while increased intragroup correlation is regarded as eligible. To study the clinical value of the m7G-based subtyping, clinical pathological features, including age, gender, and stage, were analyzed among the different subtypes. Moreover, the difference in OS among the different subtypes was explored using R packages “survival” and “survminer” (Qi-Dong et al., 2020; Li et al., 2021b).

### Biological processes and immune infiltration in different subtypes

Gene set variation analysis (GSVA) (Hänzelmann et al., 2013) was performed using R packages “limma,” “GSEABase,” “GSVA,” and “pheatmap” to study whether the biological processes among different LUAD subtypes are different. In addition, single-sample gene set enrichment analysis (ssGSEA) was conducted to estimate the immune infiltration using R packages “reshape2,” “ggpubr,” “limma,” “GSEABase,” and “GSVA” (Wang et al., 2021).

### Identification of differentially expressed genes and functional enrichment analysis

DEGs among the three LUAD subtypes were screened using the R package “limma” following p.adj < 0.001 and then combined and visualized on a Venn diagram using the package “VennDiagram.” Detailed information of DEGs is presented in Supplementary Table S2. Packages “clusterProfiler,” “org.Hs.eg.db,” “enrichplot,” and “ggplot2” were applied to explore the potential biological functions and signaling pathways of the DEGs (Xia et al., 2022b).

### Construction and validation of m7G-Riskscore

DEGs were processed for univariate regression analysis using R packages “limma” and “survival” to screen genes of prognostic significance (*p* < 0.05). Then, the genes identified were used to divide LUAD samples into different gene-clusters using the package “ConsensusClusterPlus.” All LUAD samples (*n* = 1,289) were equally grouped into training (*n* = 645) and test (*n* = 644) sets. The training set was applied to construct a prognostic model associated with m7G. During the modeling, the genes screened *via* univariate analysis were successively analyzed in LASSO regression model to eliminate overfitting and multivariate regression model to identify the most significant prognostic DEGs. The m7G-Riskscore was accordingly established and formulated as follows (Yu et al., 2015):

$$\text{m}7\text{G}-\text{Riskscore}=\sum_{\text{i}-1}^{\text{n}}\left(\text{coefi}\times \text{m}7\text{Gexpi}\right)$$

where coef represents the multivariate regression coefficient and m7Gexp represents the expression of each gene involved in the model. On the basis of the m7G-Riskscore, patients in the training set (*n* = 645) were scored and grouped with the threshold set as the median value. Survival analysis and principal component analysis (PCA) were performed using R packages “survival” and “survminer,” respectively. Distribution of the m7G-Riskscore and expression of the model genes in two groups were visualized using the R package “pheatmap.” ROC curves were plotted using the package “timeROC” (Bai et al., 2021). In parallel, samples in the total (*n* = 1,289), test (*n* = 644), and GSE30219 (*n* = 85) sets were processed for the same analysis, and corresponding graphs were generated. Moreover, uni- and multivariate analyses were performed to study the independence of the m7G-Riskscore in the prognosis of LUAD using the package “survival.” Subgroup analysis was also devised based on the clinical pathological features of LUAD in an attempt to explore whether the m7G-Riskscore remains to be powerful in prognosis in different subgroups.

### Immune infiltration, cancer stemness, mutation, and drug sensitivity analysis

The CIBERSORT algorithm (Chen et al., 2018) was calculated to estimate the abundance of 23 immune infiltrates in high- and low-risk groups. The ESTIMATE algorithm was applied to obtain stromal, immune, and ESTIMATE scores (Wu et al., 2021). The association of immune infiltrates with the model genes was investigated. In addition, cancer stemness between the two groups was analyzed. Moreover, somatic mutation in the two groups was explored, and the results were visualized *via* a waterfall plot using the R package “maftools.” At last, therapeutic response in two groups was investigated using the half-maximal inhibitory concentration (IC50) of multiple agents.

### Establishment and validation of nomogram for clinical prediction

According to the Cox regression analysis, the variables with significant prognostic values were included and combined to establish a nomogram for clinical prediction. Each patient was conferred a score, which was predictive for the 1-, 3-, and 5-year OS. Furthermore, a calibration curve was generated to assess the consistency between the predicted result and the real clinical result. Packages “survival,” “regplot,” and “rms” were adopted.

### Cell culture

Human normal lung cell line MRC-5 was cultured in MEM supplemented with 10% fetal bovine serum (FBS), whereas LUAD cell line A549 was maintained in F12K medium containing 10% FBS. The culture environment was an incubator with 5% CO_2_ and a temperature of 37°C. After routine culture, the two cell lines were further cultured on a pretreated round cover slip until 60–70% confluency was reached. Afterward, the medium was absorbed. The cells were washed with PBS thrice, fixed with 4% paraformaldehyde, and finally washed again with PBS thrice.

### Western blot and immunofluorescence staining

MRC-5 and A549 cells were collected using a cell scraper, followed by total protein extraction with an addition of cell lysis buffer (R0010, Solarbio). The protein concentration was determined using the BCA Protein Assay Kit (PC0020-50T, Solarbio). Western blot assay was applied to examine the protein levels of RRM2 (1:1000, A3424), SLC2A1 (1:1000, A11727), and CAND1 (1:1000, A14287). For immunofluorescence staining, decidual cells were obtained *via* 10-min culture with a permeabilization reagent. Dulbecco’s phosphate-buffered saline (DPBS) washing was performed thrice. After 15 min of digestion, the cells were washed thrice with DPBS and then blocked for 30 min. Excessive solution was absorbed. Primary antibodies, including RRM2 (1:500, 11661-1-AP), SLC2A1 (1:200, PB9435), and CAND1 (1:500, 100869-T10), were added overnight, followed by DPBS washing thrice after rewarming. Secondary Antibody DyLight 488-labeled goat anti-rabbit (1:100, BA1127) was subsequently added for further incubation. After 45 min, the cells were stained with DAPI staining solution (AR1176) at room temperature for 3 min. DPBS washing was achieved four times. At last, an antifade mounting medium (AR1109) was used to seal the slides, and a fluorescence microscope was applied to observe the results and take photos.

## Results

### Genetic alterations of m7G-associated genes in lung adenocarcinoma

**FIGURE 2:**
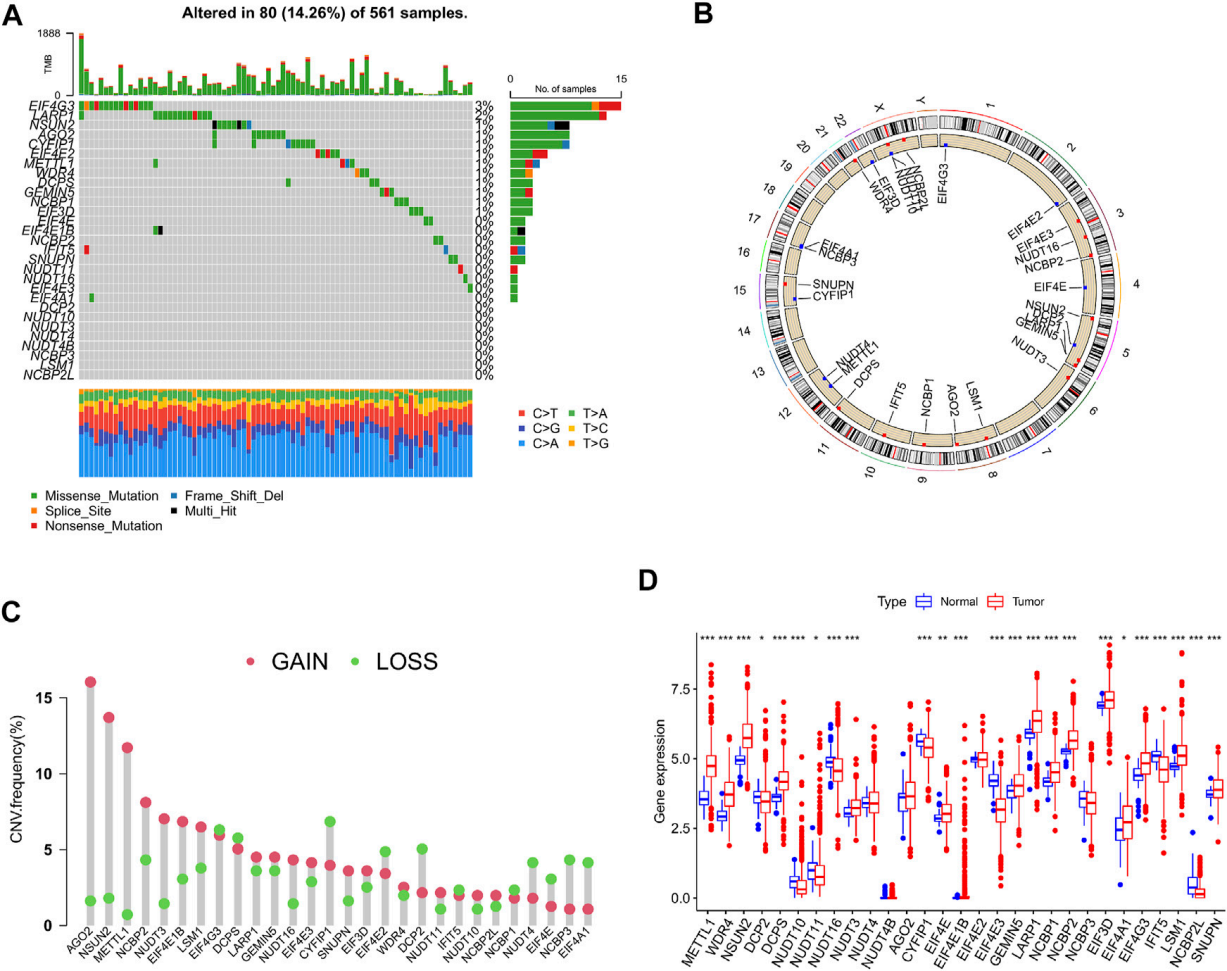
Genetic alterations of m7G-associated genes in lung adenocarcinoma (LUAD). **(A)** Mutation frequency of 29 m7G-associated genes in LUAD patients (*n* = 561). **(B)** Position of copy number variations (CNV) of the 29 m7G-associated genes on 23 chromosomes (red indicates gain of copy number; blue indicates loss of copy number). **(C)** CNV of the 29 m7G-associated genes (red indicates gain of copy number; green indicates loss of copy number). **(D)** Expression of the 29 m7G-associated genes in LUAD and normal tissue samples.

A total of 29 m7G-associated genes were included in this study and initially processed for somatic mutation analysis. The results revealed that 80 (14.26%) out of 561 LUAD patients experienced mutations, which were most frequent in the EIF4G3 gene, followed by LARP1, NSUN2, and AGO2. No mutations were found in the EIF4E and EIF4E1B genes. Missense mutation was the most common among all mutation types (Figure 2A). Copy number variations (CNV) were detected in almost all m7G-associated genes, except for EIF4E1B and NUDT4B. In the meantime, they were ubiquitous on the whole chromosomes apart from chromosomes 7, 13, 14, 18, 19, and 20. Increased copy number was demonstrated in 16 genes (such as AGO2, NSUN2, and METTL1), whereas decreased copy number was indicated in 11 genes (such as CYFIP1, EIF4G3, and DCPS) (Figures 2B, C). To clarify the association of somatic mutation with the expression of m7G-related genes, gene expression in LUAD and normal tissues was examined. It was found that partial genes exhibited a positive or negative association with CNV, whereas some genes showed no differential expression despite the increase in copy number (Figure 2D). In sum, the results revealed that CNV might be one of the regulators of the expression of m7G-associated genes.

### m7G-Associated lung adenocarcinoma subtypes and clinical prognosis

**FIGURE 3:**
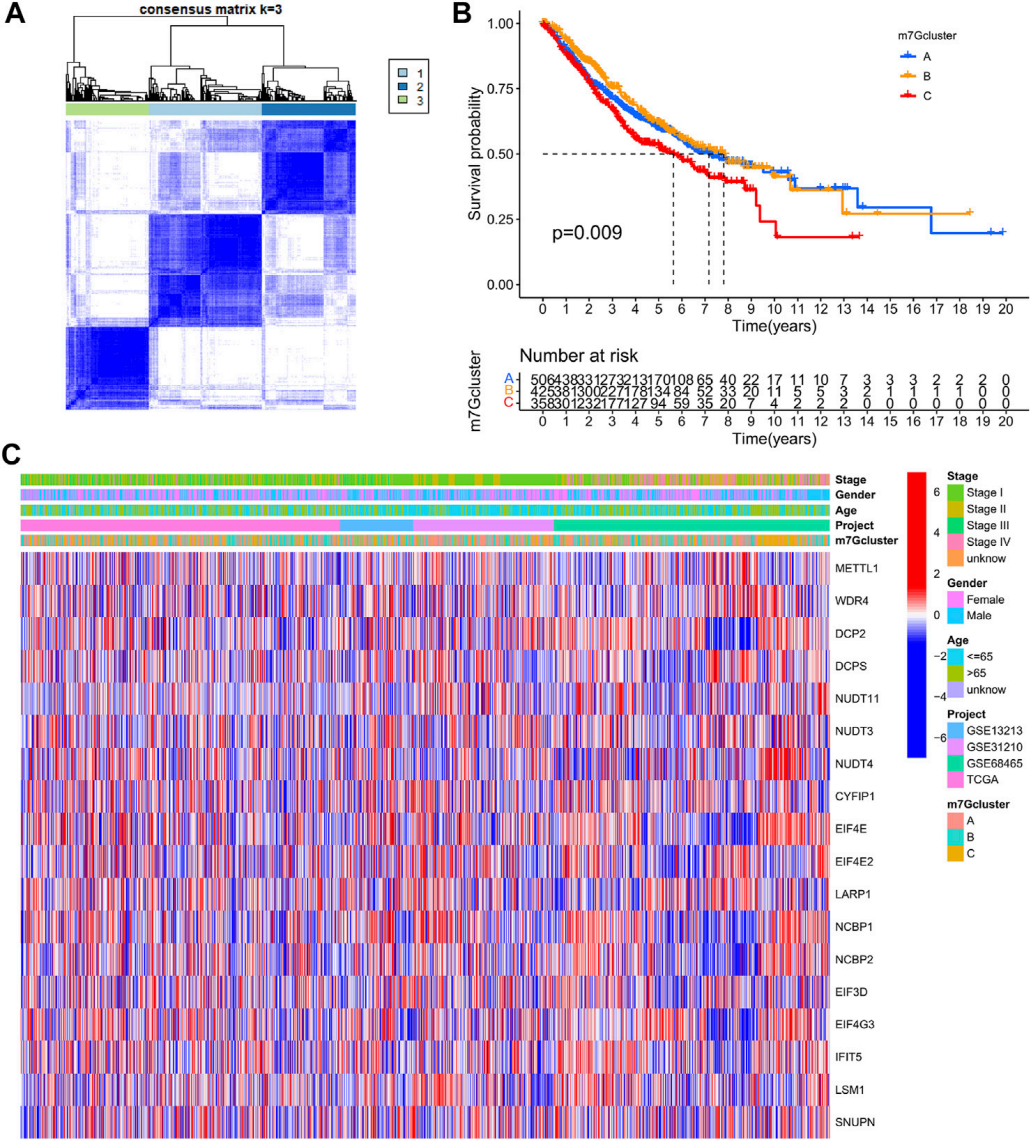
m7G-Associated LUAD subtypes and clinical prognosis. **(A)** Consensus clustering was used to divide the LUAD patients into 3 clusters (*k* = 3). **(B)** Kaplan–Meier OS curves of the 3 LUAD m7G-Clusters. **(C)** Clinical pathological features of the 3 LUAD m7G-Clusters.

LUAD data derived from TCGA-LUAD, GSE13213, GSE31210, and GSE68465 were obtained and combined. Cox regression model and survival analysis were used to assess the prognostic value of the 29 m7G-associated genes for the OS of LUAD patients. The results demonstrated that 18 out of the 29 m7G-associated genes correlated to the OS of LUAD patients (Supplementary Table S3, Supplementary Figure S1). Based on the expression of the 18 genes, consensus clustering was performed to classify LUAD patients into 3 clusters following *k* = 3 (m7G-Clusters A, B, and C) (Figure 3A, Supplementary Figure S2). Survival analysis indicated that the OS of patients in m7G-Clusters A (*p* = 0.009) and B (*p* < 0.05) was better than that of patients in m7G-Cluster C (Figure 3B). The clinical pathological features of the three m7G-Clusters are shown in Figure 3C.

### Biological and tumor microenvironment differences among the three lung adenocarcinoma subtypes

**FIGURE 4:**
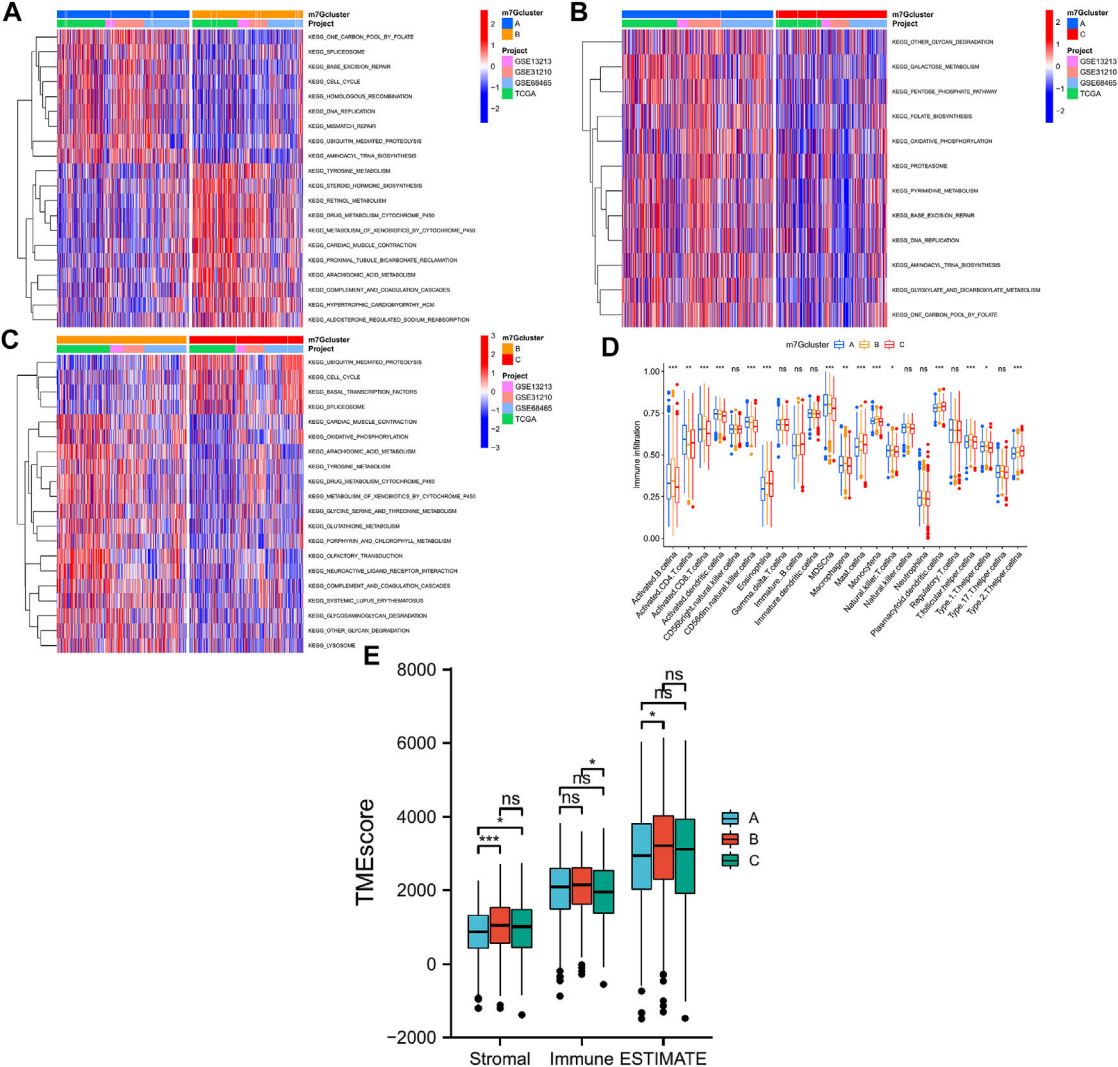
Biological processes and tumor immune microenvironment in 3 LUAD m7G-Clusters. **(A–C)** GSVA scores. **(D)** Infiltration of 23 immune cell subtypes. **(E)** Stromal, immune, and ESTIMATE scores.

GSVA was performed to explore potential significant biological pathways in the three LUAD m7G-Clusters. Proliferation-related pathways exhibited significant enrichment scores in m7G-Clusters A and C, including pathways involved in cell cycle, DNA replication, ubiquitin-mediated proteolysis, and spliceosome. In parallel, the significantly enriched pathways in m7G-Cluster B were involved in metabolism (such as metabolism of xenobiotics by cytochrome P450, drug metabolism by cytochrome P450, arachidonic acid metabolism, oxidative phosphorylation, and sphingolipid metabolism), circulation (such as olfactory transduction and cardiac muscle contraction), and immunity (such as complement and coagulation cascades) (Figures 4A–C).

In addition, the TME difference in the three LUAD m7G-Clusters was analyzed by estimating the infiltration abundance of 23 immune cell subtypes using the CIBERSORT algorithm. The results demonstrated that there were 15 immune cell subtypes with a significant differential infiltration among the three m7G-Clusters (Figure 4D). In particular, Activated.B.cellna, Activated.CD8.T.cellna, Activated.dendritic.cellna, CD56dim.natural.killer.cellna, MDSCna, Macrophagena, Monocytena, Natural.killer.T.cellna, T.follicular.helper.cellna, and Type.1.T.helper.cellna were much more abundant in m7G-Clusters A and B, whereas Plasmacytoid.dendritic.cellna and Type.2.T.helper.cellna were highly abundant in m7G-Cluster C. However, m7G-Cluster B had a significantly lower infiltration level of Activated.CD4.T.cellna than m7G-Clusters A and C. Moreover, the ESTIMATE algorithm was used to estimate the stromal, immune, and ESTIMATE scores of each cluster. The stromal score was significantly different in m7G-Cluster A vs. m7G-Cluster B and m7G-Cluster A vs. m7G-Cluster C and the immune score varied significantly between m7G-Cluster B and m7G-Cluster C, whereas the ESTIMATE score was distinct between m7G-Cluster A and m7G-Cluster B (all *p* < 0.05, Figure 4E). These results revealed that the TME of m7G-Cluster B had the highest score among the three clusters.

### Identification of m7G-associated differentially expressed genes and genotyping

**FIGURE 5:**
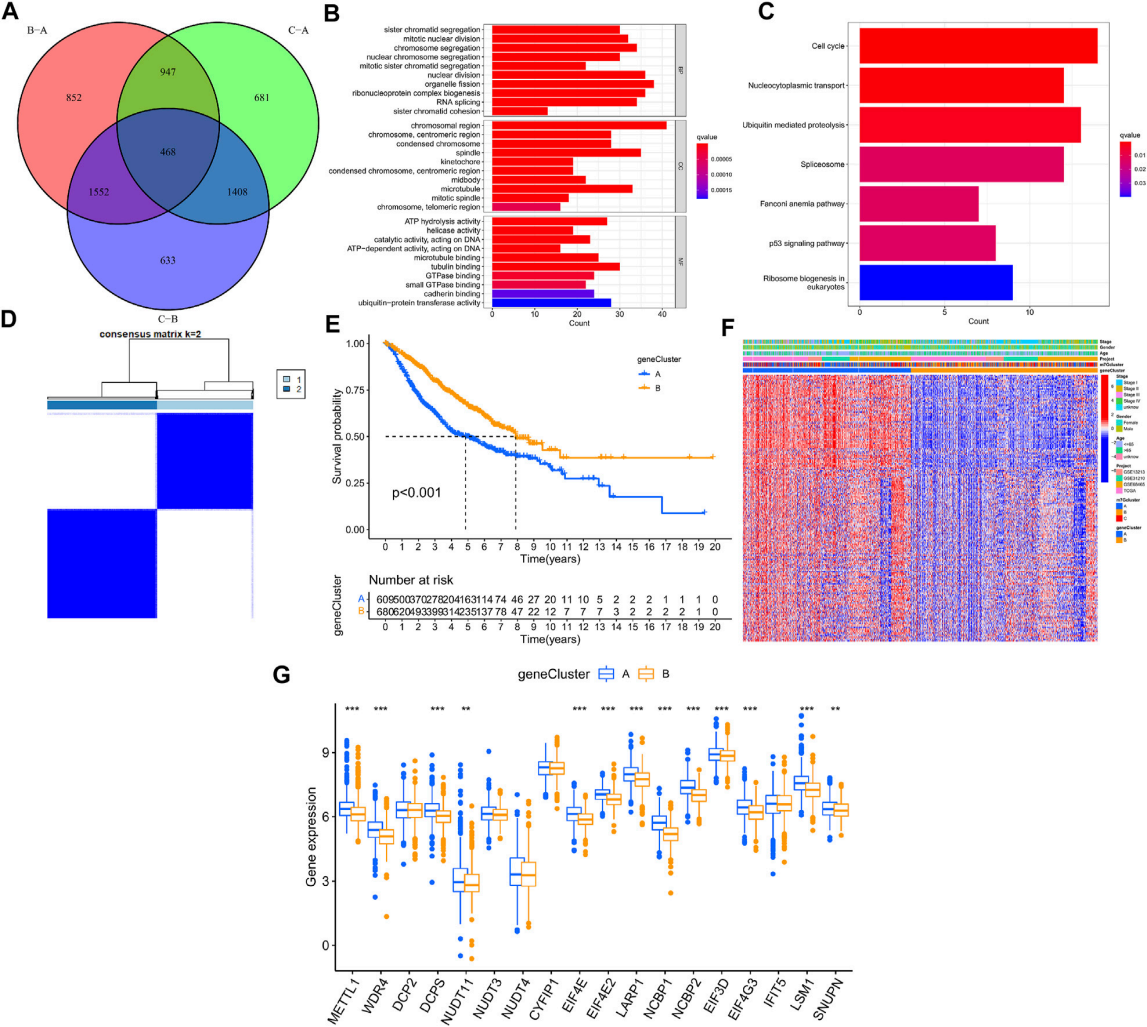
Genotyping based on m7G-associated DEGs in LUAD. **(A)** Venn diagram of DEGs from three LUAD m7G-Clusters. **(B–C)** GO and KEGG enrichment analysis for the 468 DEGs. **(D)** Consensus clustering was used to divide the LUAD patients into 2 gene clusters. **(E)** Kaplan–Meier OS curves of patients in the 2 gene clusters. **(F)** Clinical pathological features of the 2 gene clusters. **(G)** Expression of m7G-associated genes between the 2 gene clusters.

The R package “limma” was used to perform differential analysis, which resulted in the identification of 468 DEGs from the 3 LUAD m7G-Clusters (p.adj < 0.001) as shown in the Venn diagram (Figure 5A, Supplementary Table S2). The DEGs obtained were processed for functional enrichment analysis. The most enriched GO entries were biological processes involved in cell proliferation (Figure 5B), whereas the significantly enriched KEGG pathways were related to cancer cell proliferation, invasion, and death (Figure 5C). The results indicated that m7G played a vital part in tumor cell proliferation, invasion, and death. Thereafter, the 468 DEGs were subjected to univariate analysis, and 196 of them were found to be associated with the OS of LUAD (*p* < 0.05, Supplementary Table S4). Based on the 196 DEGs, consensus clustering was performed to divide the LUAD patients into two gene clusters (A, B) following *k* = 2 (Figure 5D). Further survival analysis demonstrated that patients in gene cluster B had better OS than patients in gene cluster A (*p* < 0.001, Figure 5E). In addition, gene cluster A was related to higher disease stages (III/IV) (Figure 5F). A significant difference in the expression of m7G-associated genes, such as METTL1, WDR4, and DCP2, was demonstrated between the two gene clusters (Figure 5G).

### Construction and validation of m7G-Riskscore

**FIGURE 6:**
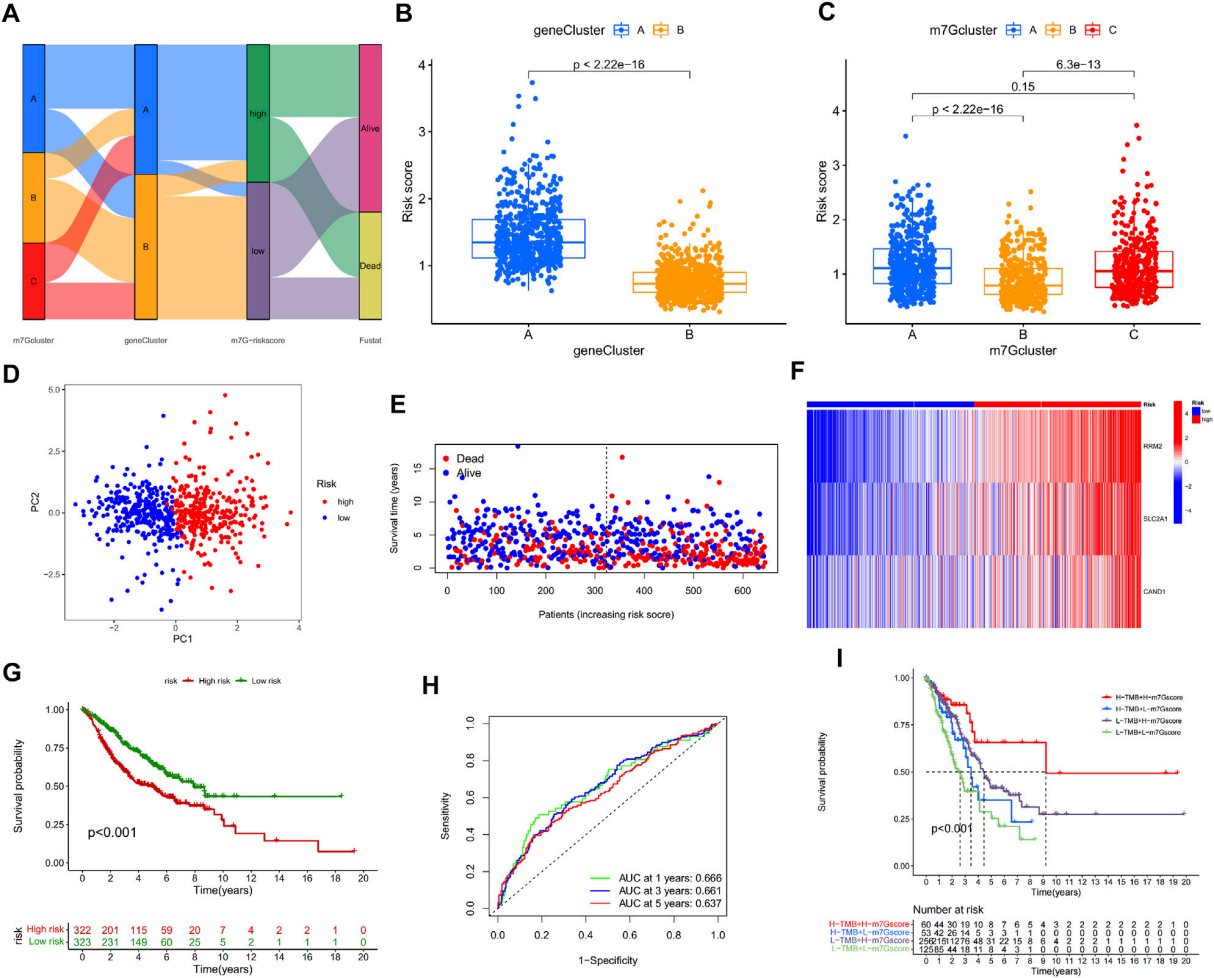
Construction and validation of m7G-Riskscore. **(A)** Sankey diagram of the clinical outcomes of patients in three m7G-Clusters, two gene clusters, and high- and low-risk groups. **(B)** m7G-Riskscore in two gene clusters. **(C)** m7G-Riskscore in 3 m7G-Clusters. **(D)** PCA. **(E)** Distribution of m7G-Riskscore. **(F)** Heatmap of the expression of the 3 signature genes (RRM2, SLC2A1, and CAND1). **(G)** Kaplan–Meier OS curves. **(H)** ROC curves for 1-, 3-, and 5-year OS. **(I)** Kaplan–Meier OS curves for LUAD patients stratified using m7G-Riskscore plus TMB.

On the basis of the three m7G-Clusters, an m7G-Riskscore was established. The information of patients stratified by m7G-associated genes, DEGs, and m7G-Riskscore is presented in Figure 6. LUAD patients were randomly categorized into training (*n* = 645) and test (*n* = 644) sets. LASSO and multivariate Cox regression models were used to analyze the 196 DEGs identified in the univariate analysis (Supplementary Figure S3, Supplementary Table S4), and 3 of them, namely, RRM2, SLC2A1, and CAND1, were selected to construct an m7G-associated prognostic signature. The m7G-Riskscore was calculated as follows: m7G-Riskscore=(0.2141×RRM2exp)+(0.1377×SLC2A1exp)+(0.2240×CAND1exp) .

Significantly higher scores were noted in gene cluster A than in gene cluster B. In addition, the scores in m7G-Clusters A and C were much higher than those in m7G-Cluster B. The findings collectively implied that the patients in m7G-Cluster B and gene cluster B had higher immune activities than those in the other groups (Figures 6B, C). Moreover, two groups of patients were defined based on the median m7G-Riskscore and validated to be well differentiated through PCA (Figure 6D). As shown in the distribution plot of the score in two groups, LUAD patients tended to survive shorter and die with an increase in the m7G-Riskscore (Figure 6E). The expression of the three signature genes in the two groups was visualized using a heatmap (Figure 6F). The patients in the high-risk group suffered from a lower survival rate than the patients in the low-risk group (*p* < 0.001, Figure 6G). The ROC curves of the m7G-Riskscore for 1-, 3-, and 5-year survival were generated, and the AUC scores were 0.666, 0.661, and 0.637, respectively, demonstrating that the m7G-Riskscore was capable of predicting the survival of LUAD patients (Figure 6H). Furthermore, the m7G-Riskscore and TMB were combined to study their effect on the survival of LUAD patients. The results showed that high TMB + high m7G-Riskscore was associated with the highest survival rate, suggesting that high TMB contributed to more immune antigens, making patients benefit more from immunotherapy (Figure 6I).

At last, the performance of the m7G-Riskscore was validated in the total, test, and GSE30219 sets. Using the same grouping strategy, LUAD patients in both sets were respectively divided into the high- and low-risk groups. The grouping quality was evaluated *via* PCA. The distributions of the m7G-Riskscore in both sets revealed that patients with a higher m7G-Riskscore had a shorter survival time. In addition, the survival rate of high-risk patients was much lower than that of low-risk patients in both the total, test, and GSE30219 sets (Supplementary Figures S4A–E, 5A–E, 6A–E). Furthermore, the ROC–AUC scores for 1-, 3-, and 5-year survival were 0.660, 0.660, and 0.629 in the total set; 0.651, 0.658, and 0.618 in the test set; and 0.974, 0.794, and 0.793 in the GSE30219 set, respectively (Supplementary Figures S4F, 5F, 6F). Taken together, the m7G-Riskscore is a robust tool with good prognostic performance for determining the OS of LUAD patients (Supplementary Figures S7, S8).

### Protein expression of the 3 m7G-associated signature genes

**FIGURE 7:**
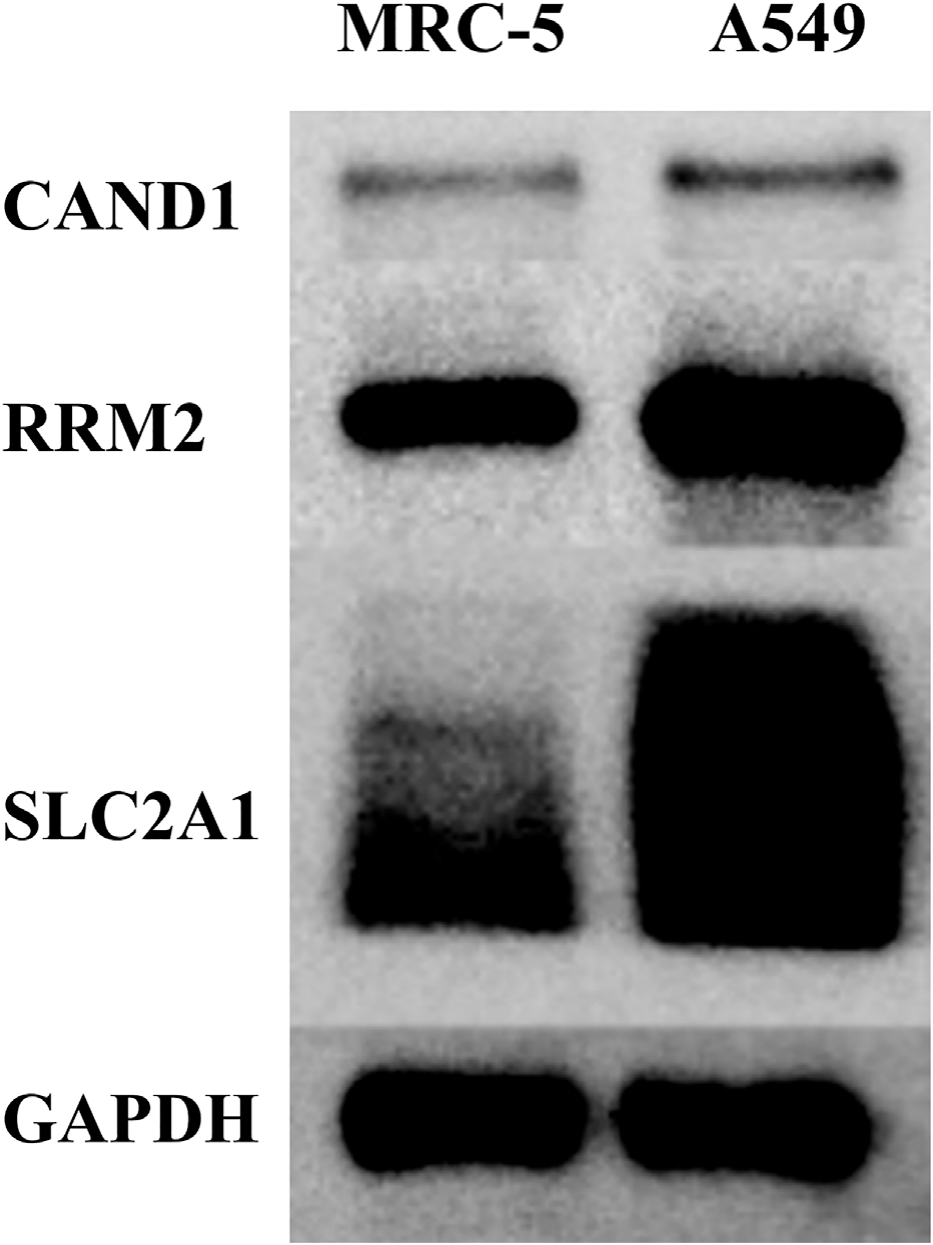
Western blot.

**FIGURE 8:**
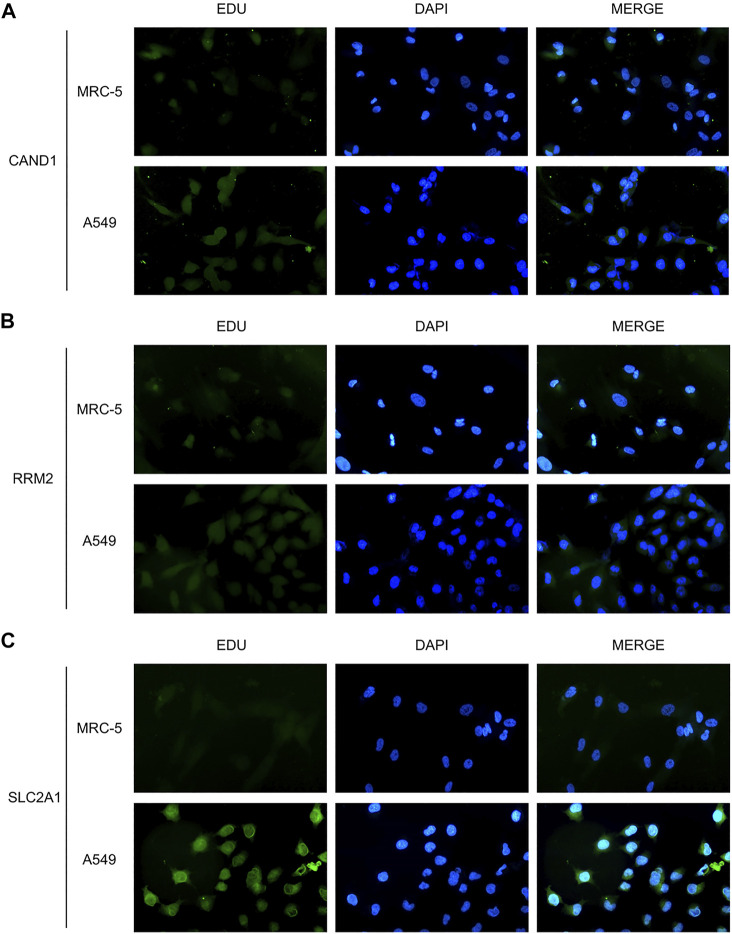
Immunofluorescence staining. **(A)** CAND1. **(B)** RRM2. **(C)** SLC2A1.

Western blot was adopted to examine the protein levels of RRM2, SLC2A1, and CAND1. As shown in Figure 7, a distinctly increased expression was found in LUAD cell line A549 than in human normal lung cell line MRC-5. In addition, immunofluorescence staining was performed and revealed the same expression trend of the three genes (Figures 8A–C).

### Tumor microenvironment of the high- and low-risk groups

**FIGURE 9:**
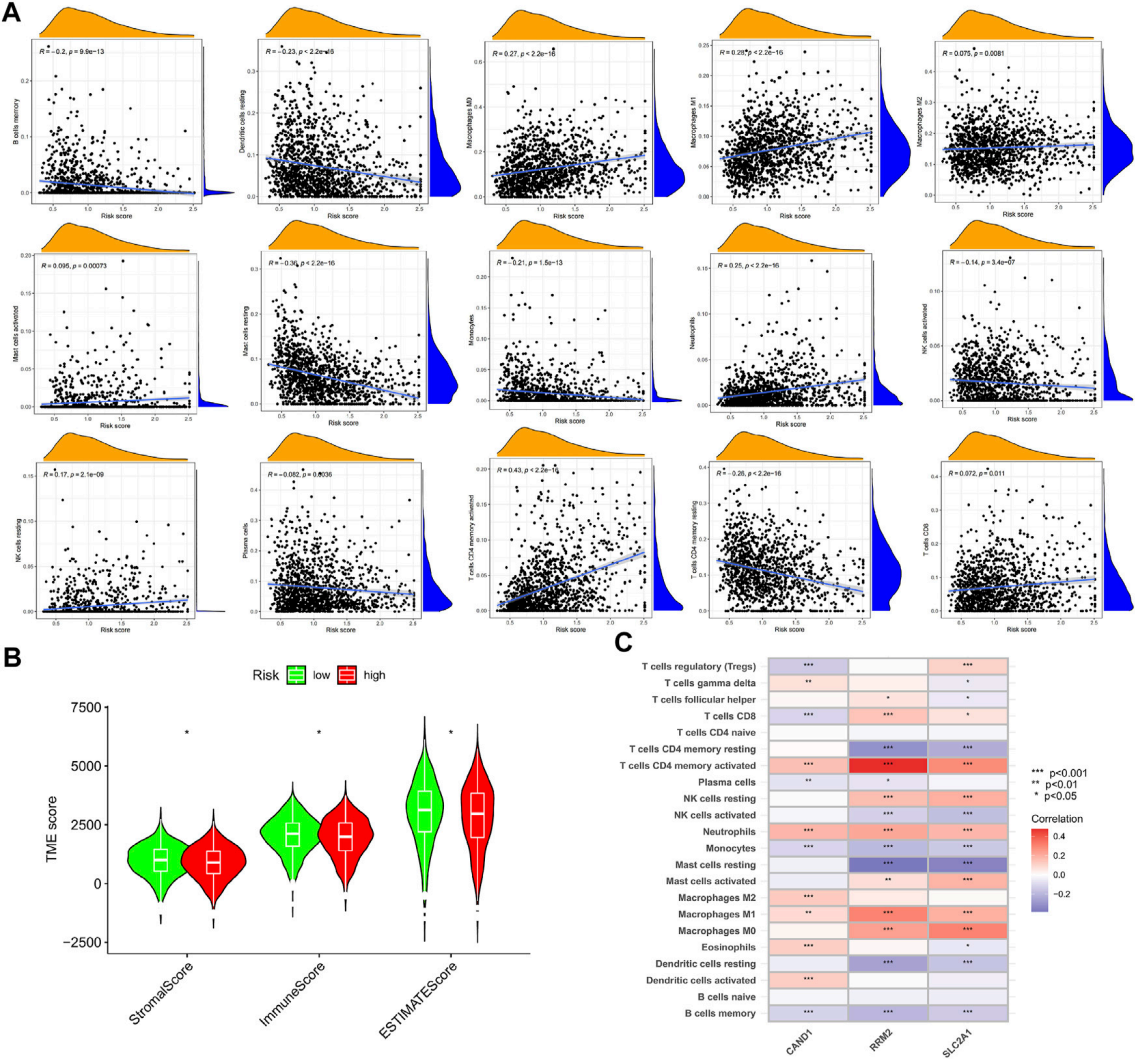
TME of the high- and low-risk groups. **(A)** Association of the m7G-Riskscore with immune infiltrates. **(B)** Association of the m7G-Riskscore with immune, stromal, and ESTIMATE scores. **(C)** Association of the three signature genes (CAND1, RRM2, and SLC2A1) with immune infiltrates.

The CIBERSORT algorithm was used to calculate the abundance of immune infiltration. It was found that the m7G-Riskscore was associated with the majority of immune infiltrates, including macrophages M0, macrophages M1, macrophages M2, mast cells activated, neutrophils, NK cells resting, CD4 memory T cells activated, and T cells CD8 (Figure 9A). In particular, negative associations were observed with the infiltration abundance of B cell memory, dendritic cells resting, mast cells resting, monocytes, NK cells activated, plasma cells, and CD4 memory T cell resting (Figure 9A). In addition, the m7G-Riskscore was noted to be negatively correlated with the immune and stromal scores (Figure 9B). Furthermore, correlation analysis revealed that expression of the three signature genes (CAND1, RRM2, and SLC2A1) was significantly associated with most of the immune infiltrates, especially CD4 memory T cells activated and neutrophils (*p* < 0.001, Figure 9C).

### Cancer stemness, mutation, and drug sensitivity in high- and low-risk groups

**FIGURE 10:**
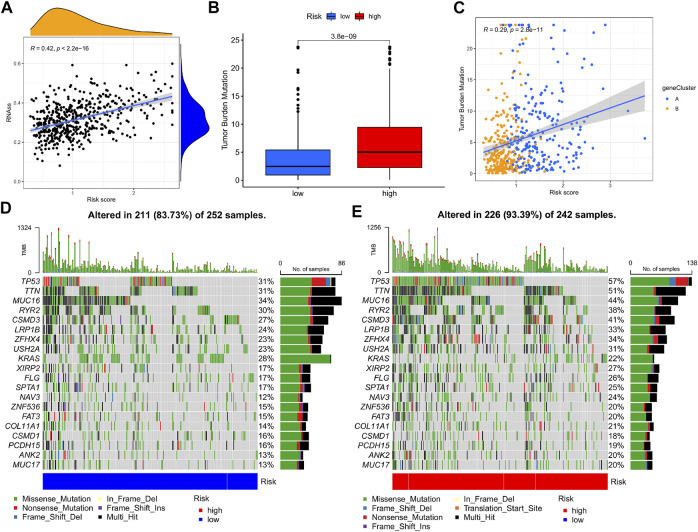
Cancer stemness and mutation analysis. **(A)** Association of the m7G-Riskscore with cancer stemness. **(B)** Association of the m7G-Riskscore with TMB. **(C)** Spearman’s correlation between the m7G-Riskscore and TMB. **(D–E)** Waterfall plots of somatic mutation.

A comprehensive analysis was devised to study the association of the m7G-Riskscore with cancer stemness, and a linear positive correlation was revealed (*R* = 0.42, *p* < 2.2e-16; Figure 10A). Research reported that TMB is conducive to the prediction of patient response to tumor immunotherapy. Here, we investigated the correlation between the m7G-Riskscore and TMB and found that the TMB increased with the increase in the m7G-Riskscore (Figure 10B). This result demonstrated that patients with a high m7G-Riskscore might benefit from immunotherapy. Furthermore, Spearman’s correlation analysis revealed a positive association (*R* = 0.29, *p* = 2.8e-11; Figure 10C). Somatic mutation was then analyzed in the TCGA-LUAD dataset. As analyzed, TP53, TTN, MUC16, RYR2, and CSMD3 exhibited the highest frequency of mutation (>25%) (Figures 10D, E). When comparing the high- and low-risk groups, the rate of somatic mutation was much higher in the high-risk group.

**FIGURE 11:**
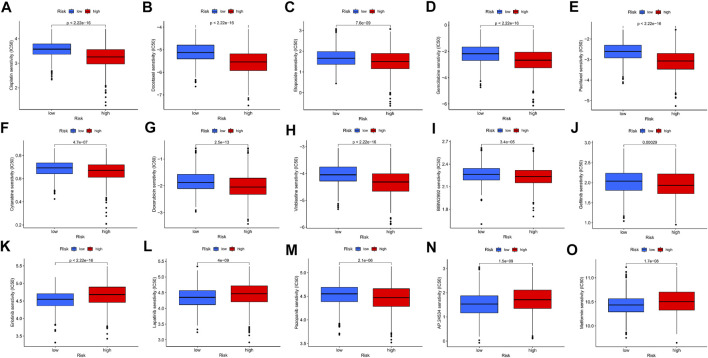
Association of the m7G-Riskscore with drug sensitivity.

To further explore the ability of the m7G-Riskscore to predict patient response to immunotherapy, the half-maximal inhibitory concentration (IC50) of multiple agents was calculated. Active responses to chemotherapeutics (such as cisplatin, docetaxel, etoposide, and gemcitabine) and multiple targeted drugs (such as afatinib, gefitinib, and erlotinib) were found in patients in the low-risk group, whereas active responses to lapatinib and metformin were observed in patients in the high-risk group (Figures 11A–O). Taken together, it was revealed that m7G-Riskscore is associated with drug sensitivity in LUAD patients.

### Establishment and validation of nomogram for clinical prediction

**FIGURE 12:**
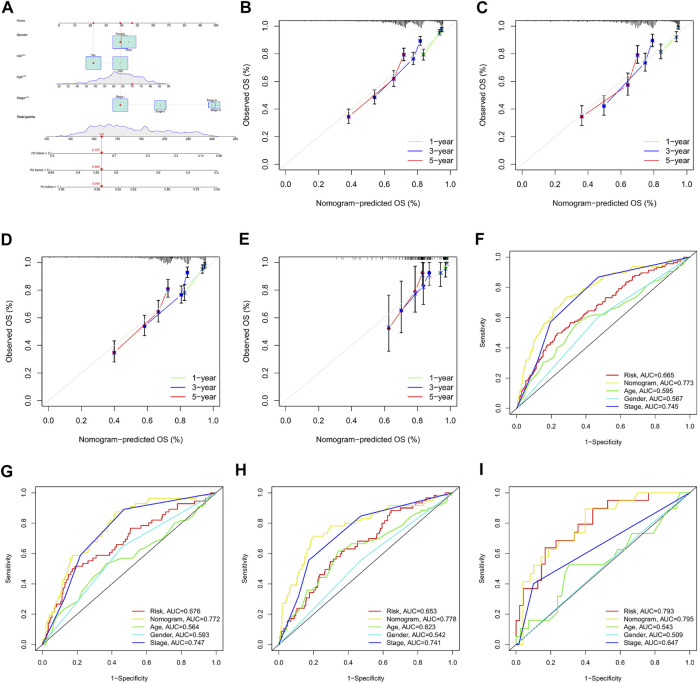
Establishment and validation of nomogram for clinical prediction. **(A)** Nomogram establishment. **(B–E)** Calibration curves for 1-, 3-, and 5-year OS in the total, training, test, and GSE30219 sets. **(F–I)** ROC curves for 1-, 3-, and 5-year OS in the total, training, test, and GSE30219 sets.

A nomogram was established based on the m7G-Riskscore and clinical pathological features (including age, gender and stage) of LUAD to predict the 1-, 3- and 5-year OS of patients (Figure 12A). Calibration curve was made to show a high consistency between the predicted result and real result in the total, training, test and GSE30219 sets, demonstrating the favorable prognostic performance of the model (Figures 12B–E). In addition, ROC–AUC scores for 1-, 3- and 5-year OS were estimated and reached the optimal as 0.773, 0.772, 0.778, and 0.795, respectively (Figures 12F–I). The results showed that the nomogram was powerful in prediction of the survival outcome of LUAD patients.

## Discussion

Owing to the increasing studies on RNA methylation, m7G methylation has shown potential in the regulation of tumor. However, majority of the studies on m7G methylation in tumor regulation focus on single or two associated genes (Chen et al., 2021; Dai et al., 2021; Orellana et al., 2021). At present, growing evidence has proven that TME is important for tumor progression and that immune infiltrates are promising prognostic factors (Pitt et al., 2016). The multimediating effects of the combination of multiple m7G-associated genes and their role in TME need to be further studied. Research reported that m7G modification was oncogenic in lung cancer (Ma et al., 2021). In particular, it is necessary to clarify the specific effect of m7G modification on lung cancer and on tumor immune infiltration.

In the present study, we initially revealed the transcriptional expression pattern of m7G-associated genes in the TCGA-LUAD dataset. Then, prognostic m7G-associated genes were screened and used to divide LUAD patients into three m7G-Clusters *via* consensus clustering. It was found that patients in m7G-Cluster C had the worst OS than those in m7G-Clusters A and B. In addition, significant differences among the three m7G-Clusters were observed with respect to biological processes, including cell proliferation, metabolism, and immunity. The TME also significantly varied in the three m7G-Clusters. Afterward, DEGs from the three m7G-Clusters were further obtained. Functional enrichment analysis revealed that the DEGs were mainly involved in pathways associated with cancer cell proliferation, invasion, and death. On the basis of the DEGs, two gene clusters were defined, whereas gene cluster B was indicative of better OS than gene cluster A. In addition, the DEGs were processed for regression analysis to obtain prognostic genes. Three genes were identified, namely, RRM2, SLC2A1, and CAND1, and used to establish an m7G-Riskscore. Each patient was conferred an m7G-Riskscore and had poorer OS when classified as gene cluster A and scored higher. This finding demonstrated that a high m7G-Riskscore was prognostic for the poor OS of LUAD patients. It was reported that m7G-associated genes are involved in the proliferation, metastasis, and drug resistance of multiple tumors (Dai et al., 2021; Xia et al., 2021), including LUAD (Ma et al., 2021). The present study found that m7G-associated genes were significantly enriched in pathways involved in cancer cell proliferation and invasion. The protein expression of the three signature genes was examined *via ex vivo* cell experiments using western blot and immunofluorescence staining assays. Multivariate analysis revealed that the m7G-Riskscore was significantly associated with the OS of LUAD patients and could be used as an independent prognostic factor. Furthermore, the ROC–AUC scores demonstrated the potential of the m7G-Riskscore in the prognosis of LUAD.

This study also found that there were significant differences in the OS, CNV, TME, cancer stemness, TMB, and drug sensitivity among high- and low-risk patients stratified using the m7G-Riskscore. Previous study showed that a higher TMB was suggestive of a better prognosis (Samstein et al., 2019), which is consistent with the present study. TME is composed of immune and stromal cells, and their scores are highly associated with the clinical features and prognosis of tumor (Quail and Joyce, 2013; Belli et al., 2018). At present, LUAD patients still have an unsatisfied prognosis and tend to develop drug resistance after routine chemotherapy (Johnson and Patel, 2014). However, the prognosis of each patient is still unpredicted despite the great advancement of targeted and immune therapies, which might be due to the varying TMEs. In the present study, we found that the m7G-Riskscore was associated with the majority of immune infiltrates, and a lower score predicted higher immune and stromal scores. The results demonstrated that the TME might play an important role in the regulation of tumor cell proliferation, invasion, and progression by m7G. In addition, a higher m7G-Riskscore suggested a higher TMB, showing that patients who scored higher may benefit more from immunotherapy. Furthermore, drug sensitivity was assessed in the high- and low-risk groups. It was found that low-risk patients responded well to chemotherapeutics and multiple targeted drugs, whereas high-risk patients better responded to lapatinib and metformin. These findings may help formulate clinical strategy in drug use and thus reduce drug resistance. At last, we established a nomogram for clinical use by combining the m7G-Riskscore and several clinical pathological features. Taken together, the m7G-associated prognostic signature we constructed could be used to stratify the prognosis of LUAD patients, help understand the molecular mechanism underlying the initiation and progression of LUAD, and provide new thoughts into cancer treatment.

The study still has some limitations. For example, the data included in this study were derived from public databases, resulting in certain selection bias and thus affecting the results. In addition, further *in vivo* experiments are required to validate the findings of the study, although we have preliminarily proven the good prognostic performance of the m7G-Riskscore and examined the *in vitro* expression of the three signature genes. Moreover, there are missing data for clinical variables, such as surgery and targeted therapy, which requires improvement and introduction of more related clinical variables to further validate the clinical value of m7G-Riskscore.

## Conclusion

To sum up, the present study systemically analyzed m7G-associated genes and revealed the related regulatory mechanism involved in the TME, pathological features, and prognosis of LUAD patients. The findings of this study demonstrated the clinical value of m7G-associated genes and provided a new thought for the clinical individualized treatment of LUAD.

## Data Availability

Publicly available datasets were analyzed in this study. This data can be found here: https://portal.gdc.cancer.gov/ and https://www.ncbi.nlm.nih.gov/geo/.

## References

[B1] Bai H. Wang Y. Liu H. Lu J. (2021). Development of a four-mRNA expression-based prognostic signature for cutaneous melanoma. Front. Genet. 12, 680617. 10.3389/fgene.2021.680617 PubMed Abstract | 10.3389/fgene.2021.680617 | Google Scholar 34335689 PMC8320537

[B2] Belli C. Trapani D. Viale G. D'Amico P. Duso B. A. Della Vigna P. (2018). Targeting the microenvironment in solid tumors. Cancer Treat. Rev. 65, 22–32. 10.1016/j.ctrv.2018.02.004 PubMed Abstract | 10.1016/j.ctrv.2018.02.004 | Google Scholar 29502037

[B3] Bi Y. Xiang D. Ge Z. Li F. Jia C. Song J. (2020). An interpretable prediction model for identifying N^7^-methylguanosine sites based on XGBoost and SHAP. Mol. Ther. Nucleic Acids 22, 362–372. 10.1016/j.omtn.2020.08.022 PubMed Abstract | 10.1016/j.omtn.2020.08.022 | Google Scholar 33230441 PMC7533297

[B4] Chen B. Khodadoust M. S. Liu C. L. Newman A. M. Alizadeh A. A. (2018). Profiling tumor infiltrating immune cells with CIBERSORT. Methods Mol. Biol. 1711, 243–259. 10.1007/978-1-4939-7493-1_12 PubMed Abstract | 10.1007/978-1-4939-7493-1_12 | Google Scholar 29344893 PMC5895181

[B5] Chen Z. Zhu W. Zhu S. Sun K. Liao J. Liu H. (2021). METTL1 promotes hepatocarcinogenesis via m^7^ G tRNA modification-dependent translation control. Clin. Transl. Med. 11 (12), e661. 10.1002/ctm2.661 PubMed Abstract | 10.1002/ctm2.661 | Google Scholar 34898034 PMC8666584

[B6] Dai Z. Liu H. Liao J. Huang C. Ren X. Zhu W. (2021). N^7^-Methylguanosine tRNA modification enhances oncogenic mRNA translation and promotes intrahepatic cholangiocarcinoma progression. Mol. Cell 81 (16), 3339–3355. 10.1016/j.molcel.2021.07.003 PubMed Abstract | 10.1016/j.molcel.2021.07.003 | Google Scholar 34352206

[B7] Hänzelmann S. Castelo R. Guinney J. (2013). GSVA: Gene set variation analysis for microarray and RNA-seq data. BMC Bioinform. 14, 7. 10.1186/1471-2105-14-7 10.1186/1471-2105-14-7 | Google Scholar PMC3618321 23323831

[B8] Johnson M. L. Patel J. D. (2014). Chemotherapy and targeted therapeutics as maintenance of response in advanced non-small cell lung cancer. Semin. Oncol. 41 (1), 93–100. 10.1053/j.seminoncol.2013.12.007 PubMed Abstract | 10.1053/j.seminoncol.2013.12.007 | Google Scholar 24565583

[B9] Li R. Di L. Li J. Fan W. Liu Y. Guo W. (2021). A body map of somatic mutagenesis in morphologically normal human tissues. Nature 597 (7876), 398–403. 10.1038/s41586-021-03836-1 PubMed Abstract | 10.1038/s41586-021-03836-1 | Google Scholar 34433965

[B10] Li R. Yin Y. H. Ji X. L. Liu X. Li J. P. Qu Y. Q. (2021). Pan-cancer prognostic, immunity, stemness, and anticancer drug sensitivity characterization of N6-methyladenosine RNA modification regulators in human cancers. Front. Mol. Biosci. 8, 644620. 10.3389/fmolb.2021.644620 PubMed Abstract | 10.3389/fmolb.2021.644620 | Google Scholar 34150845 PMC8211991

[B11] Ma J. Han H. Huang Y. Yang C. Zheng S. Cai T. (2021). METTL1/WDR4-mediated m^7^G tRNA modifications and m^7^G codon usage promote mRNA translation and lung cancer progression. Mol. Ther. 29 (12), 3422–3435. 10.1016/j.ymthe.2021.08.005 PubMed Abstract | 10.1016/j.ymthe.2021.08.005 | Google Scholar 34371184 PMC8636169

[B12] Martincorena I. Campbell P. J. (2015). Somatic mutation in cancer and normal cells. Science 349 (6255), 1483–1489. 10.1126/science.aab4082 PubMed Abstract | 10.1126/science.aab4082 | Google Scholar 26404825

[B13] Milholland B. Auton A. Suh Y. Vijg J. (2015). Age-related somatic mutations in the cancer genome. Oncotarget 6 (28), 24627–24635. 10.18632/oncotarget.5685 PubMed Abstract | 10.18632/oncotarget.5685 | Google Scholar 26384365 PMC4694783

[B14] Orellana E. A. Liu Q. Yankova E. Pirouz M. De Braekeleer E. Zhang W. (2021). METTL1-mediated m^7^G modification of Arg-TCT tRNA drives oncogenic transformation. Mol. Cell 81 (16), 3323–3338. 10.1016/j.molcel.2021.06.031 PubMed Abstract | 10.1016/j.molcel.2021.06.031 | Google Scholar 34352207 PMC8380730

[B15] Pitt J. M. Marabelle A. Eggermont A. Zitvogel L. (2016). Targeting the tumor microenvironment: removing obstruction to anticancer immune responses and immunotherapy. Ann. Oncol. 27 (8), 1482–1492. 10.1093/annonc/mdw168 PubMed Abstract | 10.1093/annonc/mdw168 | Google Scholar 27069014

[B16] Qi-Dong X. Yang X. Lu J. L. Liu C. Q. Sun J. X. Li C. (2020). Development and validation of a nine-redox-related long noncoding RNA signature in renal clear cell carcinoma. Oxid. Med. Cell. Longev. 2020, 6634247. 10.1155/2020/6634247 PubMed Abstract | 10.1155/2020/6634247 | Google Scholar 33425212 PMC7781722

[B17] Quail D. F. Joyce J. A. (2013). Microenvironmental regulation of tumor progression and metastasis. Nat. Med. 19 (11), 1423–1437. 10.1038/nm.3394 PubMed Abstract | 10.1038/nm.3394 | Google Scholar 24202395 PMC3954707

[B18] Samstein R. M. Lee C. H. Shoushtari A. N. Hellmann M. D. Shen R. Janjigian Y. Y. (2019). Tumor mutational load predicts survival after immunotherapy across multiple cancer types. Nat. Genet. 51 (2), 202–206. 10.1038/s41588-018-0312-8 PubMed Abstract | 10.1038/s41588-018-0312-8 | Google Scholar 30643254 PMC6365097

[B19] Shiba-Ishii A. (2021). Significance of stratifin in early progression of lung adenocarcinoma and its potential therapeutic relevance. Pathol. Int. 71 (10), 655–665. 10.1111/pin.13147 PubMed Abstract | 10.1111/pin.13147 | Google Scholar 34324245

[B20] Succony L. Rassl D. M. Barker A. P. McCaughan F. M. Rintoul R. C. (2021). Adenocarcinoma spectrum lesions of the lung: detection, pathology and treatment strategies. Cancer Treat. Rev. 99, 102237. 10.1016/j.ctrv.2021.102237 PubMed Abstract | 10.1016/j.ctrv.2021.102237 | Google Scholar 34182217

[B21] Sung H. Ferlay J. Siegel R. L. Laversanne M. Soerjomataram I. Jemal A. (2021). Global cancer statistics 2020: GLOBOCAN estimates of incidence and mortality worldwide for 36 cancers in 185 countries. CA Cancer J. Clin. 71 (3), 209–249. 10.3322/caac.21660 PubMed Abstract | 10.3322/caac.21660 | Google Scholar 33538338

[B22] Tomikawa C. (2018). 7-Methylguanosine modifications in transfer RNA (tRNA). Int. J. Mol. Sci. 19 (12), 4080. 10.3390/ijms19124080 10.3390/ijms19124080 | Google Scholar PMC6320965 30562954

[B23] Wang X. Pan L. Lu Q. Huang H. Feng C. Tao Y. (2021). A combination of ssGSEA and mass cytometry identifies immune microenvironment in muscle-invasive bladder cancer. J. Clin. Lab. Anal. 35 (5), e23754. 10.1002/jcla.23754 PubMed Abstract | 10.1002/jcla.23754 | Google Scholar 33813769 PMC8128294

[B24] Wilkerson M. D. Hayes D. N. (2010). ConsensusClusterPlus: a class discovery tool with confidence assessments and item tracking. Bioinformatics 26 (12), 1572–1573. 10.1093/bioinformatics/btq170 PubMed Abstract | 10.1093/bioinformatics/btq170 | Google Scholar 20427518 PMC2881355

[B25] Wu J. Li L. Zhang H. Zhao Y. Wu S. Xu B. (2021). A risk model developed based on tumor microenvironment predicts overall survival and associates with tumor immunity of patients with lung adenocarcinoma. Oncogene 40 (26), 4413–4424. 10.1038/s41388-021-01853-y PubMed Abstract | 10.1038/s41388-021-01853-y | Google Scholar 34108619

[B26] Xia P. Zhang H. Xu K. Jiang X. Gao M. Wang G. (2021). MYC-targeted WDR4 promotes proliferation, metastasis, and sorafenib resistance by inducing CCNB1 translation in hepatocellular carcinoma. Cell Death Dis. 12 (7), 691. 10.1038/s41419-021-03973-5 PubMed Abstract | 10.1038/s41419-021-03973-5 | Google Scholar 34244479 PMC8270967

[B27] Xia Q. D. Sun J. X. Xun Y. Xiao J. Liu C. Q. Xu J. Z. (2022). SUMOylation pattern predicts prognosis and indicates tumor microenvironment infiltration characterization in bladder cancer. Front. Immunol. 13, 864156. 10.3389/fimmu.2022.864156 PubMed Abstract | 10.3389/fimmu.2022.864156 | Google Scholar 35418978 PMC8995476

[B28] Xia Q. D. Sun J. X. Liu C. Q. Xu J. Z. An Y. Xu M. Y. (2022). Ferroptosis patterns and tumor microenvironment infiltration characterization in bladder cancer. Front. Cell Dev. Biol. 10, 832892. 10.3389/fcell.2022.832892 PubMed Abstract | 10.3389/fcell.2022.832892 | Google Scholar 35386202 PMC8978677

[B29] Xie S. Chen W. Chen K. Chang Y. Yang F. Lin A. (2020). Emerging roles of RNA methylation in gastrointestinal cancers. Cancer Cell Int. 20 (1), 585. 10.1186/s12935-020-01679-w PubMed Abstract | 10.1186/s12935-020-01679-w | Google Scholar 33372610 PMC7720447

[B30] Yu Z. Chen H. You J. Liu J. Wong H. S. Han G. (2015). Adaptive fuzzy consensus clustering framework for clustering analysis of cancer data. IEEE/ACM Trans. Comput. Biol. Bioinform. 12 (4), 887–901. 10.1109/TCBB.2014.2359433 PubMed Abstract | 10.1109/TCBB.2014.2359433 | Google Scholar 26357330

[B31] Zhao Y. Kong L. Pei Z. Li F. Li C. Sun X. (2021). m7G methyltransferase METTL1 promotes post-ischemic angiogenesis via promoting VEGFA mRNA translation. Front. Cell Dev. Biol. 9, 642080. 10.3389/fcell.2021.642080 PubMed Abstract | 10.3389/fcell.2021.642080 | Google Scholar 34136476 PMC8200671

