# Supplementary material for: m7G-Associated subtypes, tumor microenvironment, and validation of prognostic signature in lung adenocarcinoma
Source: Front Genet. 2022 Aug 10;13:954840. doi: 10.3389/fgene.2022.954840 (PMC9422053; doi:10.3389/fgene.2022.954840)
Supplement: Supplementary file 1 [file DataSheet1.docx]

**Supplementary Figures**


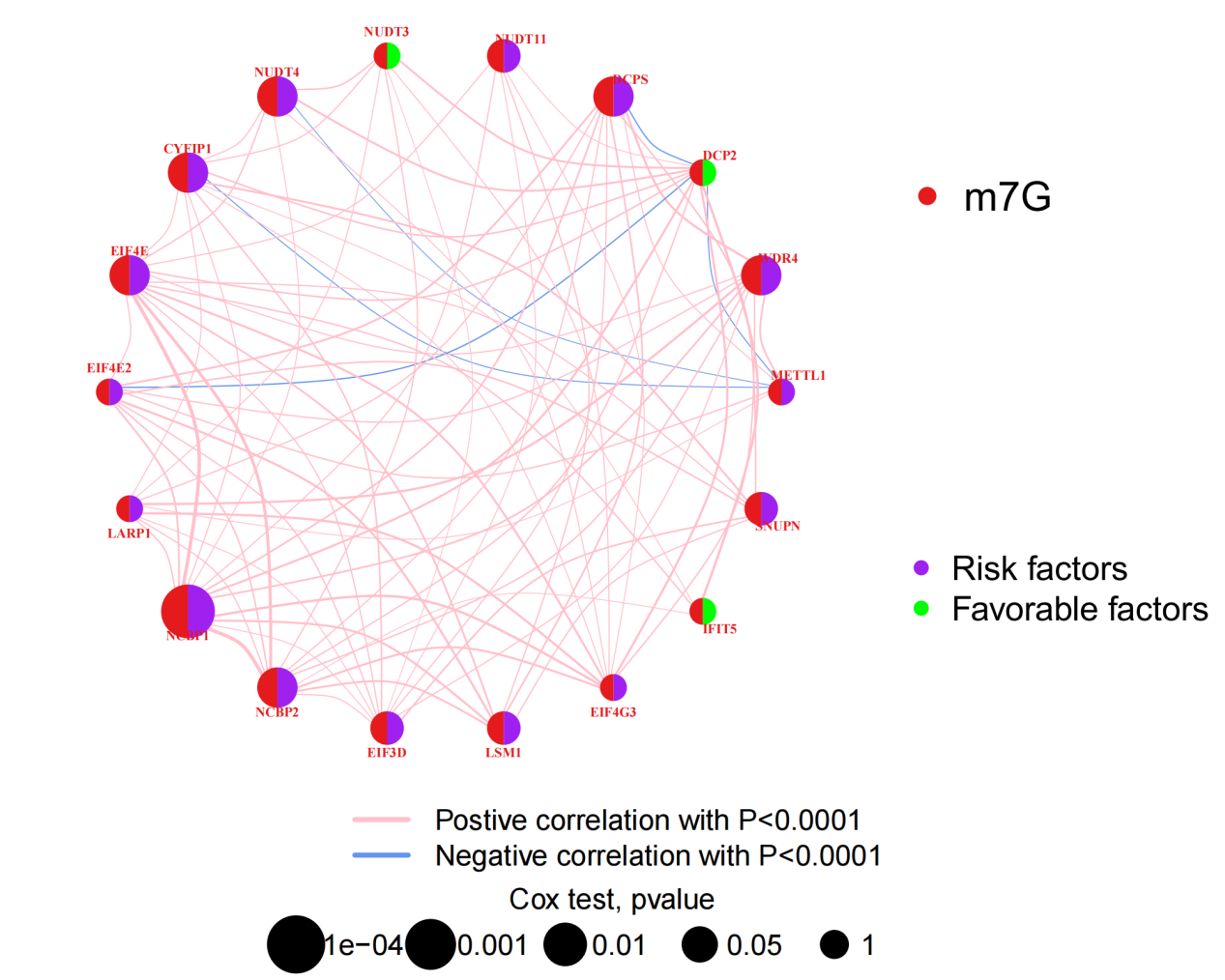


**Figure S1** Interactions among m7G-associated genes in LUAD. The line connecting the m7G-associated genes represents their interaction, with the line thickness indicating the strength of the association between m7G-associated genes. Blue and pink represent negative and pink positive correlations, respectively.


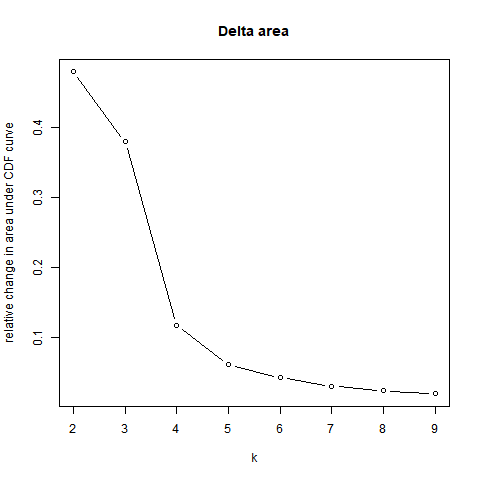


**Figure S2** The delta area score displayed the relative growth in cluster stability.


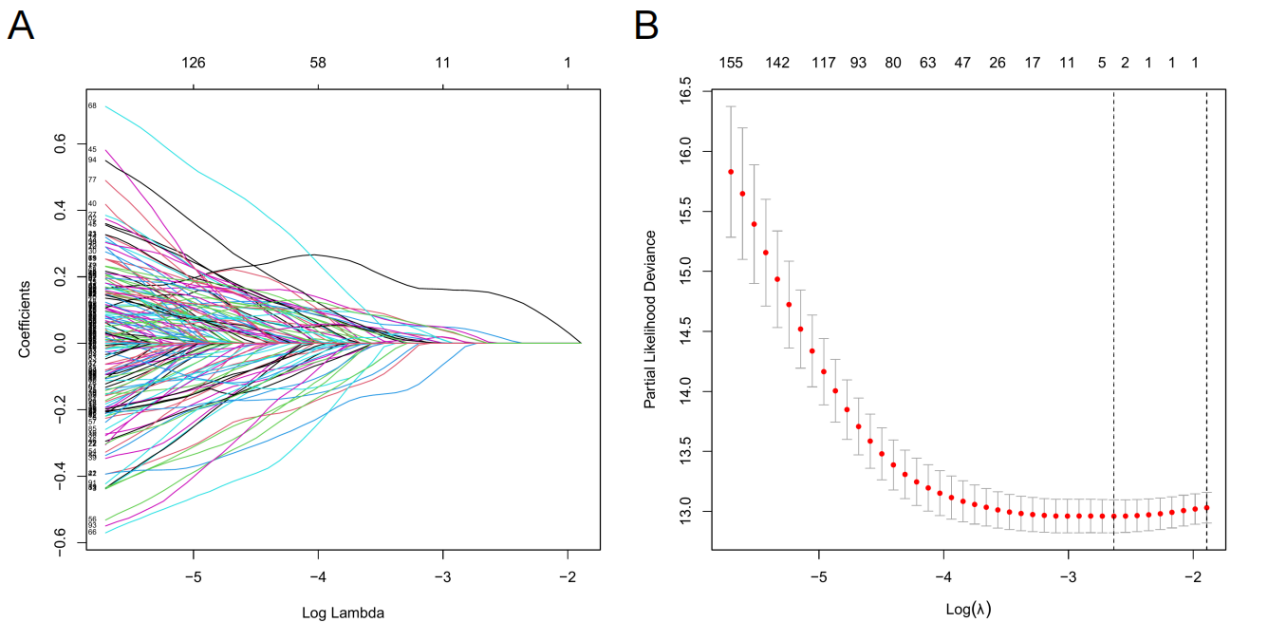


**Figure S3** Identifying representative candidate prognostic genes. (A-B) The LASSO regression analysis and partial likelihood deviance on the prognostic genes.


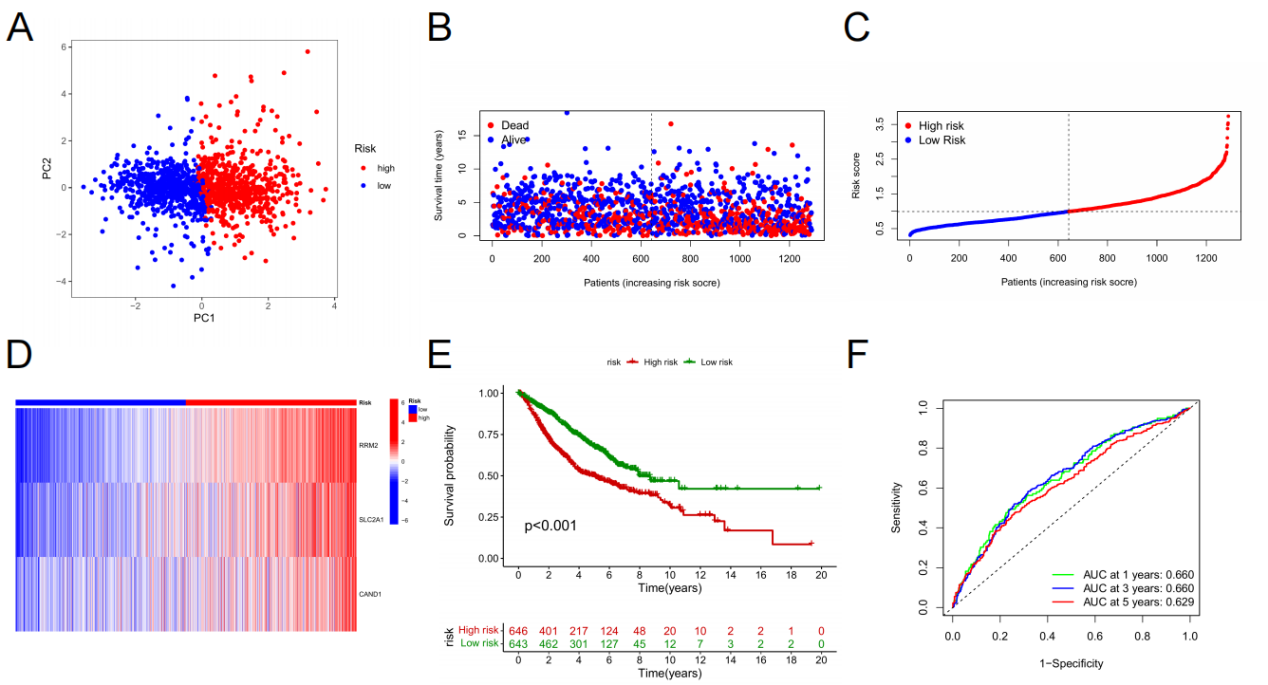


**Figure S4** Validation of m7G-Riskscore in entire cohort. (A) The PCA analysis demonstrated that the patients in the different risk groups were distributed in two directions. (B-C) Distribution of m7G-Riskscore. (D) Heatmap of expression of the 3 signature genes (RRM2, SLC2A1 and CAND1) in entire cohort. (E) KM analysis of the OS between the two groups.The ranked dot plot indicates the m7G-Riskscore distribution and scatter plot presenting the patients’ survival status. (F) ROC curves to predict the sensitivity and specificity of 1-, 3-, and 5-year survival according to the m7G-Riskscore.


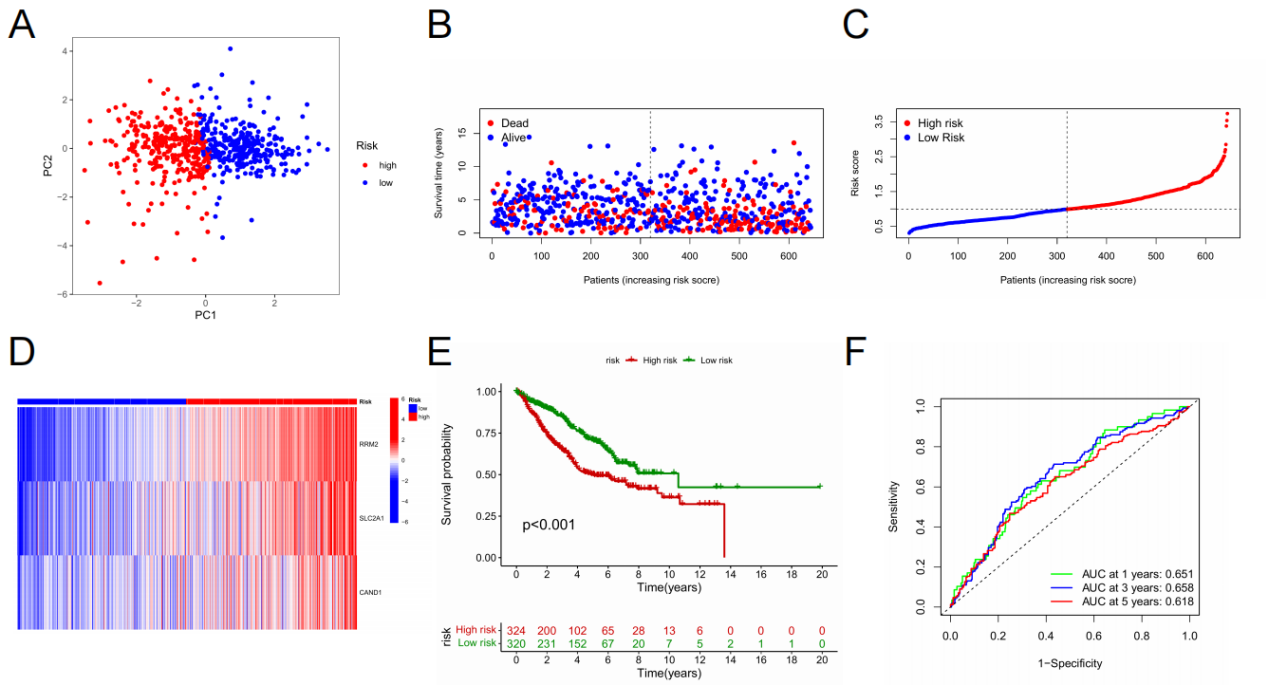


**Figure S5** Validation of m7G-Riskscore in test cohort. (A) The PCA analysis demonstrated that the patients in the different risk groups were distributed in two directions. (B-C) Distribution of m7G-Riskscore. (D) Heatmap of expression of the 3 signature genes (RRM2, SLC2A1 and CAND1) in entire cohort. (E) KM analysis of the OS between the two groups.The ranked dot plot indicates the m7G-Riskscore distribution and scatter plot presenting the patients’ survival status. (F) ROC curves to predict the sensitivity and specificity of 1-, 3-, and 5-year survival according to the m7G-Riskscore.


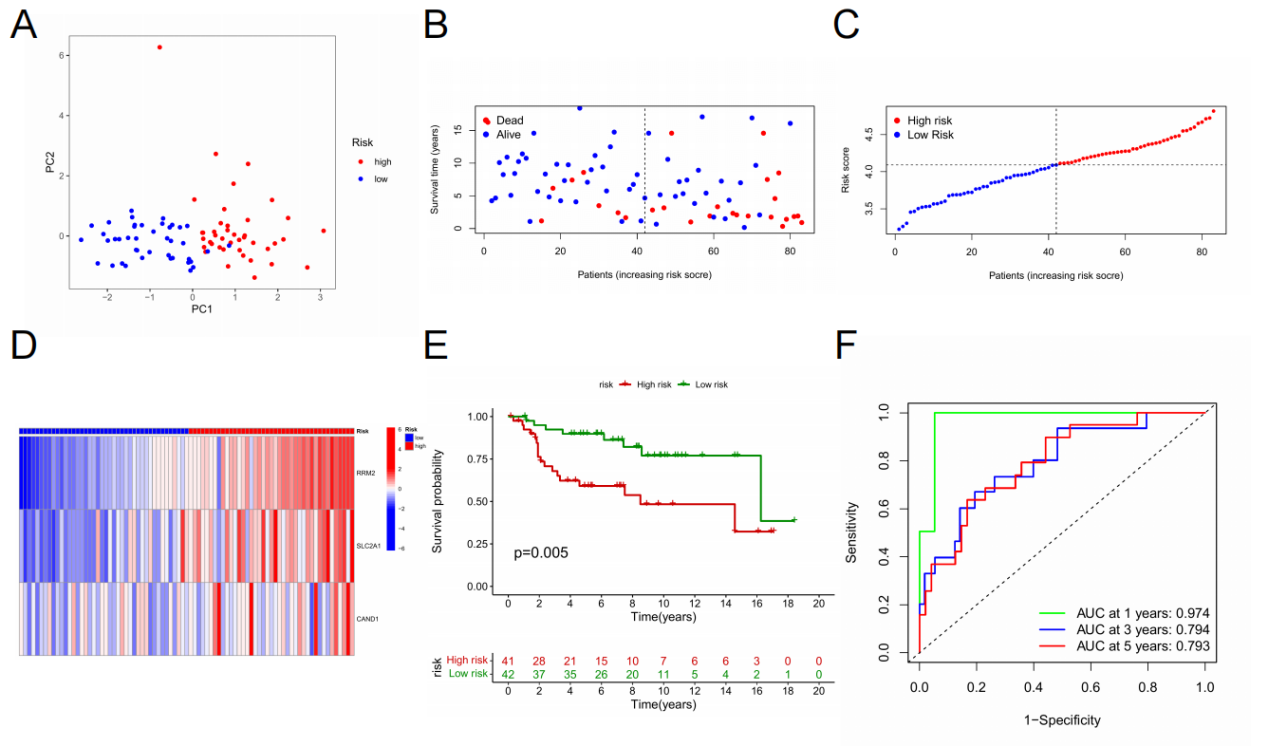


**Figure S6** Validation of m7G-Riskscore in GSE30219 cohort. (A) The PCA analysis demonstrated that the patients in the different risk groups were distributed in two directions. (B-C) Distribution of m7G-Riskscore. (D) Heatmap of expression of the 3 signature genes (RRM2, SLC2A1 and CAND1) in entire cohort. (E) KM analysis of the OS between the two groups.The ranked dot plot indicates the m7G-Riskscore distribution and scatter plot presenting the patients’ survival status. (F) ROC curves to predict the sensitivity and specificity of 1-, 3-, and 5-year survival according to the m7G-Riskscore.


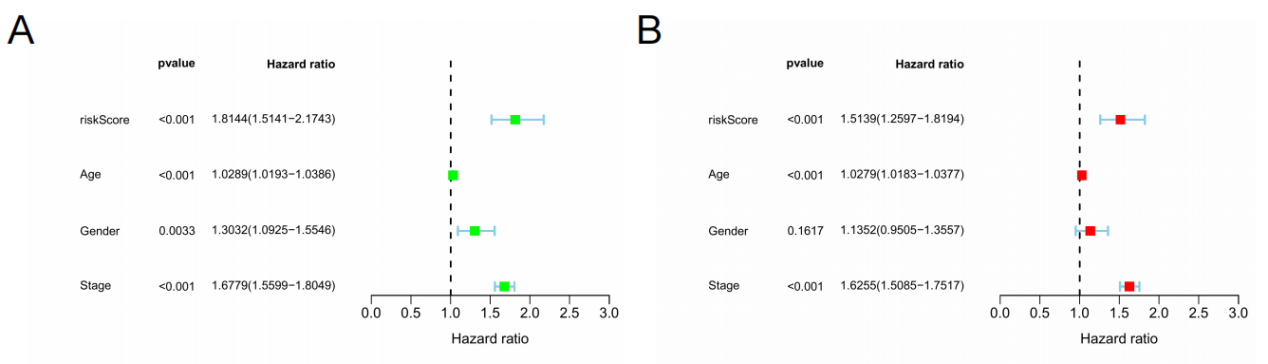


**Figure S7** The independent prognosis analysis of m7G-Riskscore and clinicopathological variables in LUAD. (A-B) Univariate and multivariate analyses showed the prognostic value of the m7G-Riskscore.


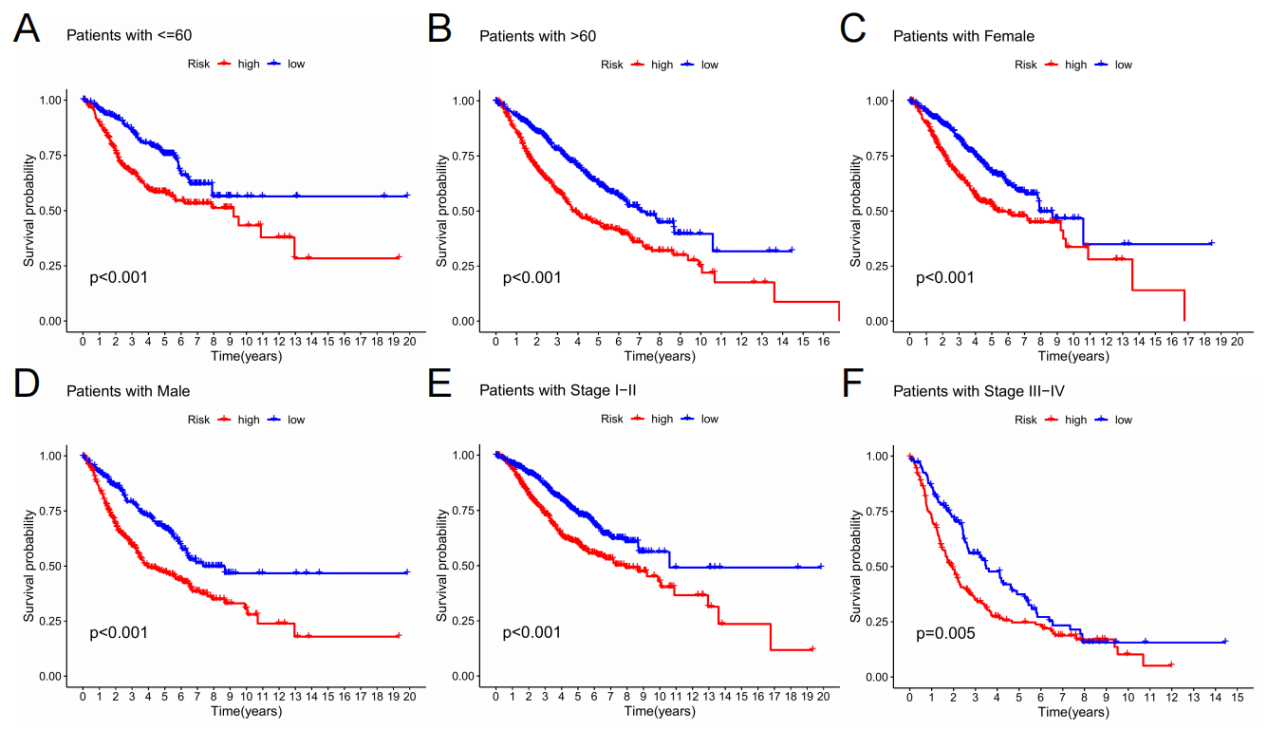


**Figure S8** Stratification analysis of the m7G-Riskscore in LUAD. (A-B) Age (age ≤ 60 and age > 60 years old). (C-D) Gender (male and female). (E-F) Stage (I-II and III-IV).
